# Diagnostic accuracy of magnetic resonance imaging techniques for treatment response evaluation in patients with high-grade glioma, a systematic review and meta-analysis

**DOI:** 10.1007/s00330-017-4789-9

**Published:** 2017-03-22

**Authors:** Bart R. J. van Dijken, Peter Jan van Laar, Gea A. Holtman, Anouk van der Hoorn

**Affiliations:** 1 University Medical Center Groningen Department of Radiology, University of Groningen, Hanzeplein 1, P. O. Box 30.001, 9700 RB Groningen, The Netherlands; 2 University Medical Center Groningen, Center for Medical Imaging-North East Netherlands, University of Groningen, Groningen, The Netherlands; 3 University Medical Center Groningen, Department of General Practice, University of Groningen, Groningen, The Netherlands

**Keywords:** Glioma, Magnetic resonance imaging, Meta-analysis, Magnetic resonance spectroscopy, Treatment response

## Abstract

**Objective:**

Treatment response assessment in high-grade gliomas uses contrast enhanced T1-weighted MRI, but is unreliable. Novel advanced MRI techniques have been studied, but the accuracy is not well known. Therefore, we performed a systematic meta-analysis to assess the diagnostic accuracy of anatomical and advanced MRI for treatment response in high-grade gliomas.

**Methods:**

Databases were searched systematically. Study selection and data extraction were done by two authors independently. Meta-analysis was performed using a bivariate random effects model when ≥5 studies were included.

**Results:**

Anatomical MRI (five studies, 166 patients) showed a pooled sensitivity and specificity of 68% (95%CI 51–81) and 77% (45–93), respectively. Pooled apparent diffusion coefficients (seven studies, 204 patients) demonstrated a sensitivity of 71% (60–80) and specificity of 87% (77–93). DSC-perfusion (18 studies, 708 patients) sensitivity was 87% (82–91) with a specificity of 86% (77–91). DCE-perfusion (five studies, 207 patients) sensitivity was 92% (73–98) and specificity was 85% (76–92). The sensitivity of spectroscopy (nine studies, 203 patients) was 91% (79–97) and specificity was 95% (65–99).

**Conclusion:**

Advanced techniques showed higher diagnostic accuracy than anatomical MRI, the highest for spectroscopy, supporting the use in treatment response assessment in high-grade gliomas.

***Key points*:**

*• Treatment response assessment in high-grade gliomas with anatomical MRI is unreliable*

*• Novel advanced MRI techniques have been studied, but diagnostic accuracy is unknown*

*• Meta-analysis demonstrates that advanced MRI showed higher diagnostic accuracy than anatomical MRI*

*• Highest diagnostic accuracy for spectroscopy and perfusion MRI*

*• Supports the incorporation of advanced MRI in high-grade glioma treatment response assessment*

**Electronic supplementary material:**

The online version of this article (doi:10.1007/s00330-017-4789-9) contains supplementary material, which is available to authorized users.

## Introduction

High-grade gliomas (HGG) are the most common primary brain tumours in adults and have low survival rates [1]. Current standard therapy consists of surgical gross total or subtotal resection followed by concomitant chemoradiotherapy (CCRT) and adjuvant chemotherapy with temozolomide (TMZ) [2]. Decisions about continuation or discontinuation of treatment for individual patients with high-grade gliomas depend on adequate imaging. Similarly, identification of new active drugs often depends on assessment of an objective response rate, which is established by changes in the tumour seen on imaging [3].

Traditionally, response assessment in HGG is done on the basis of assessment by contrast (gadolinium) enhanced T1-weighted MRI. However, this technique represents a disruption of the blood-brain barrier and thereby does not measure tumour activity specifically [4]. In many situations, changes in enhancement do not correlate with response. Up to 50% of the patients show pseudo-progression, in which an increase in contrast enhancement does not reflect tumour progression, but treatment induced changes [5].

To overcome limitations of anatomical T1-weighted MRI with gadolinium, more advanced imaging techniques have been employed in patients with HGG [4]. Diffusion-weighted MRI is frequently performed in routine clinical practice to image changes in cytoarchitecture and cellular density [6, 7]. However, even newer imaging methods based on MRI can identify tumour-induced neovascularization (perfusion weighted MRI) and changes in concentrations of metabolites (magnetic resonance spectroscopy) [6–8].

Many small limited studies have shown the potential usefulness of the different advanced techniques for assessment of treatment response in HGG [6–8]. However, a systematic review and meta-analysis demonstrating the diagnostic accuracy of the anatomical and all advanced MRI techniques is lacking.

To this end, we conducted a systematic review and meta-analysis to provide an overview of the diagnostic accuracy of treatment response assessment in HGG patients. We hypothesized that advanced MRI techniques show a higher diagnostic accuracy compared to anatomical MRI techniques in patients treated for HHG.

## Methods

This systematic review and meta-analysis was performed according to the Preferred Reporting Items for Systematic Reviews and Meta-Analysis (PRISMA) criteria [9]. Additionally, the AMSTAR guidelines and the Cochrane handbook for review of diagnostic test accuracy were also used [10].

### Search strategy

See electronic supplementary material.

### Selection criteria

Studies including HGG patients that received first line standard therapy according to the Stupp protocol and underwent anatomical or advanced MRI imaging were included [2]. Studies were included if 2x2 tables could be extracted. The definitive diagnosis, either treatment induced changes or tumour progression, was established by histological follow-up, imaging follow-up, clinical follow-up, or a combination of these.

Reasons for exclusion were other intracranial malignancies, metastases, and brainstem or optic gliomas. Studies among paediatric patients (<18 years) and case reports were also excluded. Studies that were conducted before 2005 were excluded as TMZ was not incorporated in standard therapy before 2005, while TMZ might increase the occurrence of treatment related imaging changes [7, 11]. Finally, studies that used a MRI <1.5 Tesla were excluded as this does not represent current clinical practice.

### Study selection, data extraction, and quality assessment

After duplicates were eliminated, studies were screened for eligibility based on title, abstract, and subsequently on full text by two authors independently (BD, AH). Data from the included studies were extracted with the use of a data extraction form. Extracted data contained true positives, false positives, true negatives, false negatives, and general characteristics. General characteristics included total number of patients, study design, mean age, and age range of patients, gender, tumour histology, selection criteria of included patients, reference standard (histology/imaging/clinical follow-up), MRI characteristics and time-point of progression on MRI, and the cut-off value of the index test. If 2x2 tables could not be generated, the authors were requested to provide these data. Study quality was assessed according to the quality assessment of diagnostic accuracy studies (QUADAS-2) [12].

### Statistical analysis

Sensitivity and specificity with 95% confidence interval (CI) were calculated for all MRI modalities in RevMan 5.3 (Cochrane collaboration, Copenhagen, Denmark). Analyses of study heterogeneity are not recommended, because it is a univariate measure that does not account for heterogeneity explained by phenomena such as positivity threshold effects [13]. Visual inspection of the generated forest plots was done to assess heterogeneity. We evaluated whether the following factors could explain heterogeneity; study design, mean age of patients, WHO type, cut-off value of the index test, type of follow-up, and time point of progression on MRI (see also Table 1). We performed subgroup analysis (≥5 studies) to explore and explain heterogeneity in test characteristics. Moreover, we evaluated whether outliers could be explained by study or patient characteristics, and we performed sensitivity analysis without outliers to evaluate how robust the results are.

**Table 1:** Characteristics of the included studies

Reference	*N*	Study type	Age (years) mean ± SD (range)	% male	Histology	Selection	Reference standard	Field strength; MRI technique, orientation, slice thickness/ gap in mm (TR/TE/TI in ms); b values	Time point MRI	Diagnostic accuracy (cut-off if provided in the paper)	TP	FP	TN	FN
Al Sayyari et al. [14]	16	Pros	54 (30-92)	50	WHO III: 6 WHO IV: 10	HGG with new enhan-cement after treatment	Histology (*N* = 4), radioclinical (*N* = 12)	1.5/3 T. T1 tra 5/- (500-600/7.4-11); T1C tra 5/- (500-600/7.4-11); SWI 3D (49-27/20-40); DWI tra (3900-4500/84-91) b 0 1000.	5.6 and 8.1 mo (1-26) after end treatment	ADC (ROI based on SWI) ADC (ROI based on T1C)	9 4	0 2	5 4	2 7
Alexiou et al. [15]	30	Pros	62 ± 11.1	70	WHO III: 3 WHO IV: 27	HGG with suspected recurrence on cMRI.	Histology (*N* = 2), radioclinical (*N* = 28)	1.5 T. T1 3D 1/0 (25/4.6); T1C tra, sag, cor 1/0 (25/4.6); T2 tra 6/0.6 (3000/90); FLAIR tra 6/0.6 (6300/120/2150); DWI tra 3/0 (9807/131) b 0, 700; DSC tra 7/0 (702/30).	1 mo after end RT with follow-up every 3 mo	rCBV (2.2) ADC (1.27) FA (0.47)	24 16 14	0 0 0	6 6 6	0 8 10
Baek et al. [16]	79	Retro	51 (19-83)	58	WHO IV: 79	GBM with new or enlarged enhan-cement after treatment	Histology (*N* = 22), radioclinical (*N* = 57)	3 T. T1 tra 5/- (475/10); T1C tra, cor, sag 5/- (450-495/10); T2 tra 5/- (3000/80); DWI tra 5/- (3804/48), b-; DSC tra 5/- (1407/40).	<4 w after end CCRT 4-8 w after first follow-up	Histogram: max (3.1) mode (1.6) range (2.5) %Δ skew (1.17) %Δ kurtosis (5.14) Histogram pattern (3)	39 31 33 36 26 36	10 6 6 8 10 4	27 31 31 29 27 33	3 11 9 6 16 6
Barajas et al. [17]	57	Retro	54 ± 10.2	58	WHO IV: 57	GBM after treatment	Histology (*N* = 55), imaging (*N* = 2)	1.5 T. T1 sag -/- (600/17); T1C sag -/- (1000/54); T1C 3D -/- (34/8); T2 3D -/- (3000/102); FLAIR tra -/- (10000/148/2200); DSC 5/- (1250/54)	1.7–50.2 mo after end RT	PH (1.38) rCBV (1.75) PSR (87.3%)	41 36 36	4 6 5	16 14 15	5 10 10
Bisdas et al. [18]	18	Pros	-	56	WHO III + IV: 56	HGG with suspected recurrence after treatment	Histology (*N* = 5), imaging (*N* = 13)	3 T. T1 - -/- (279/2.5); T1C - -/- (279/2.5); T1C 3D (1300/2.6); DCE 4/-(3.4/1.4).	7.8-13 mo after end CCRT, follow-up with 2-mo intervals	K^trans^ (0.19) AUC (15.35)	12 9	1 2	5 4	0 3
Cha et al. [19]	35	Retro	49 (24-70)	51	WHO IV: 35	GBM with new or enlarged enhan-cement <180 d after treatment	Histology (*N* = 3), imaging (*N* = 32),	3 T. T1C tra 5/- (500/10); DWI tra 5/- (3000/75) b 0, 1000; DSC tra 5/- (1720/35).	124 ± 34.7 d (79-204) after surgery	Size enhan-cement rCBV (1.80) CBV mode (1.60)	8 9 9	4 4 0	20 20 24	3 2 2
Choi et al. [20]	62	Retro	49 (22-79)	60	WHO IV: 62	GBM with new enhan-cement <4 w after treatment	Histology (*N* = 43), imaging (*N* = 19)	- T. T1 tra 5/- (475/10); T1C tra, cor, sag 5/- (450-495/10); T2 tra 5/- (3000/80); DWI tra 5/- (3804/46); DSC tra -/-(1407/40); ASL tra 6/- (3000/13). ASL:	MRI follow-up intervals of 2-3 mo	ASL AUC (0.774) DSC	27 28	10 9	18 19	7 6
Chung et al. [21]	57	Retro	51 (25-69)	53	WHO IV: 57	GBM after treatment.	Histology (*N* = 57)	3 T. DCE 4/0 (6.4/3.1)	40 mo after end CCRT	_m_AUCR 0.23) _90th_AUCR (0.32)	30 29	3 3	22 22	2 3
D’Souza et al. [22]	27	Pros	43 (18-61)	74	WHO III: 16 WHO IV: 11	HGG after therapy	Histology (*N* = 20), radioclinical (*N* = 7)	3 T. T1C tra -/- (2000/12); T2 tra -/- (5600/100 ms); FLAIR tra, cor -/- (9000/81/2500); DSC 4/- (1600/30); MRS single voxel 8-12 x 8-12 x8-12 (2000/30), Cho, Cr, NAA; MRS multi voxel 10 x 10 x 15 (1700/30), Cho, Cr NAA.	10 mo (7-19) after treatment	rCBV Cho/Cre (2.00)	14 14	0 1	10 9	3 3
Dandois et al. [23]	7	Retro	51 (25-74)	57*	WHO III: 1 WHO IV: 6	HGG after treatment	Histology (*N* = 2), imaging (*N* = 4), clinical (*N* = 1)	1.5 T. T1C tra 5/1 (30/3); T1C 3D 1.2/0 (30/3); T2 tra 5/1 (4390/90); FLAIR tra 5/1 (10000/120/2100); DWI tra 5/1(3312/93), b 0, 1000; DSC tra 5/1 mm (1500/35 ms).CE-T1:	-	T1C FLAIR rCBV (182%)	2 3 5	0 0 0	2 2 2	2 2 0
Di Constanzo et al. [24]	29	Pros	63 (38-74)	62	WHO IV: 29	GBM with new enhan-cement after treatment	Imaging (*N* = 29)	3 T. T1 sag 5/1 (225/ 2.5); T1C 3D 1.4/0 (225/3.2); T2 tra 5/1 (5000/85); FLAIR tra 5/1(11000/140/2250); DWI tra 5/1 mm (11000/66.6),b 0 1000; DSC 5/1 mm (1700/48 ms); MRS multivoxel 7.5 x 7.5 x 10 (1500/144);	8 w after end CCRT and with 3-mo intervals during 1st year and 3-6-mo intervals thereafter	ADC rCBV Cho NAA Cr Cho/Cr Cho/NAA	17 18 15 13 12 17 10	1 1 2 2 0 2 0	7 7 6 6 8 6 8	4 3 6 8 9 4 11
Goenka et al. [25] (abstract)	32	Pros	-	-	WHO III + IV: 32	HGG after treatment	Histology and/or radioclinical	1.5 T. DWI; PWI; MRS multivoxel Cho, NAA, Cho/Cr, NAA/Cr.	-	rCBV (2.30) DWI	15 10	6 1	15 14	0 11
Heidemans- Hazelaar et al. [26] (abstract)	32	Retro	-	-	WHO IV: 32	GBM with new lesion on cMRI after treatment	Histology or imaging	- T. PWI	-	rCBV (2.12)	25	1	5	3
Hu et al. [27]	13	Pros	48 (31-62)	85	WHO III: 4 WHO IV: 9	HGG with new enhan-cement after treatment undergoing re-resection	Histology (*N* = 13)	3 T. T1C fs 3D 2/0 (6.8/2.8/300); DSC 5/0 mm (2000/20).	-	rCBV (0.71)	22	2	16	0
Hu et al. [28]	11	Pros	47	91	WHO III: 3 WHO IV: 8	HGG with suspected recurrence after treatment undergoing re-resection	Histology (*N* = 11)	3 T. T1C fs 3D 2/0 (6.8/2.8/300); DSC 5/0 mm (2000/20).	-	rCBV without BLS/PLD (0.92-0.96) rCBV with BLS/PLD (1.02-1.03)	13 19	0 0	15 15	8 2
Jora et al. [29]	7	Pros	43 ± 14.9*	61*	WHO III + IV: 7	PHGG with suspected residual or recurrence after treatment	Histology (*N* = 7)	1.5 T. T1 tra, sag 3-5/- (400-550/14); T1C tra, cor, sag 3/- (400/15); T2 3/- (4000/126-130).	-	cMRI	3	1	2	1
Kim et al. [30]	51	Retro	52 (35-72)	49	WHO IV: 51	GBM with new or enlarged enhan-cement after treatment undergoing re-resection	Histology (*N* = 51)	3 T. DWI - -/- (-/-) b 0, 10, 20, 40, 60, 80, 100, 120, 140, 160, 180, 200, 300, 500, 700 and 900; DSC - -/-(1407/40).	12.5 d before re-resection; 44 w post CCRT	f90(0.056) D10 (0.970) nCBV_90_ (2.892) ADC_10_ (0.995)	27 22 26 21	1 5 1 5	19 15 19 15	4 9 5 10
Kong et al. [31]	90	Pros	50 (25-74)	83	WHO IV: 90	GBM with new or enlarged enhan-cement after treatment	Histology (*N* = 4), imaging (*N* = 86)	3 T. T1 - 5/1.5 (500/10); T2 - 5/1.5 (3000/80); FLAIR - 5/1.5 (11000/ 125/-); DSC - 5/2 (1500/35).	4 w after end treatment and with 2-mo intervals	rCBV (1.49)	27	6	51	6
Larsen et al. [32]	13	Pros	58 (38-75)	85	WHO III: 4 WHO IV: 9	HGG with unclear cMRI after treatment	Histology (*N* = 9), imaging (*N* = 2), clinical (*N* = 2)	3 T. DCE - 8/1.5 (3.9/1.9).	16 ± 13 mo (3-48) after end RT	DCE	11	0	2	0
Lee et al. [33]	22	Retro	49 (18-69)	64	WHO III: 3 WHO IV: 19	GBM with new enhan-cement after treatment	Imaging (*N* = 22)	- T. T1 - 5/1 (558-650/8-20); T2 - 5/1 (4500-5160/91-106.3); FLAIR 5/1 (9000-9900/97-162.9/-); DWI tra 3/1 (6900-10000/55-70) b 0, 1000.	24 d; (11-60) after end CCRT	ADC (1200x10^-6)^	8	2	10	2
Nakajima et al. [34]	12	Retro	50 (23-67)	33	WHO III: 5 WHO IV: 7	HGG with new lesion on cMRI after treatment	Histology (*N* = 11), radioclinical (*N* = 1)	1.5 T. MRS single voxel 12-20 x 12-20 x 16-20 (2000/272).	24.2 mo (4-80) after end RT	Cho/Cre (2.50) Lac/Cho (1.05)	5 5	1 0	6 7	0 0
Palumbo et al. [35]	24	Pros	53 ± 13.7 (25-76)	73*	WHO III: 8 WHO IV: 16	HGG with unclear cMRI after treatment	Histology (*N* = 24)	1.5 T. T1 sag 5/0.5 (540/18); T2 cor 5/0.5 (4000/100 ms); FLAIR tra 5/0.5 (8000/120/2000); 1MRS single voxel 4-6 cc (144/2500).	6-12 mo after surgery	MRS	16	0	7	1
Peca et al. [36]	15	Pros	53 (28-72)	45	WHO IV: 15	GBM after treatment	Histology (*N* = 10), imaging (*N* = 5)	- T. MRS	4 w after end RT	MRS	11	3	1	0
Pica et al. [37] (abstract)	26	Pros	-	-	WHO III: 10 WHO IV: 16	HGG with clinical symptoms after treatment	Histology (*N* = 8), imaging (*N* = 18)	- T. DSC	-	rCBV (3.70)	10	6	10	1
Pugliese et al. [38] (abstract)	24	Retro	-	-	WHO IV: 24	GBM after treatment	Histology or imaging	- T. DSC	<4 mo after surgery	rCBV (2.30)	8	3	9	3
Reddy et al. [39]	51	Retro	47 (22-71)	65	WHO III: 16 WHO IV: 35	GBM after treatment undergoing re-resection	Histology (*N* = 51)	- T. T1 tra, cor and/or sag - -/- (-/-); T1C tra, cor and/or sag - -/- (-/ -); T2 and/or FLAIR - /-/ (-/-).	2-11 d before re-resection; 7.3 mo after initial surgery	cMRI	17	1	17	3
Seeger et al. [40]	40	Retro	54 ± 13.6	60	WHO III + IV: 40	HGG with new enhan-cement after treatment	Imaging (*N* = 40)	1.5 T. DSC - 5/- (1610/30); DCE 3D 5/- (4/1.16); ASL - 5/- (2600/16); MRS multivoxel 10 x 10 x 15 (1570/135).	-	ASL rCBF (2.18) DCE K^trans^ K(0.058) DSC rCBF r(2.24) DSC rCBV (2.15) Cho/Cr (1.07)	12 14 18 19 16	3 3 3 4 4	14 14 14 13 13	11 9 5 4 7
Song et al. [41]	20	Retro	51 ± 13.5 (24-68)	50	WHO IV: 20	GBM with enhan-cement after treatment	Imaging (*N* = 20)	- T. T1 tra 5/1 (558-650/8-20); T2 tra 5/1 (4500-5160/91-106.3); FLAIR tra 5/1 (9000-9900/97-162.9); DWI tra 3/1 (6900-10000/55-70) b 0, 1000; DSC tra 5/1 (1500/30-40)	22 d (11-34) after end CCRT	ADC ROC curve Observer 1 Observer 2	9 8	1 2	9 8	1 2
Suh et al. [42]	79	Retro	51 (25-69)	46	WHO IV: 79	GBM with new or enlarged enhan-cement after treatment	Histology (*N* = 24), imaging with clinical progression (*N* = 55)	3 T. DCE 3D 4/0 (6.4/3.1).	<4-5 w after end CCRT	mAUCR (0.31) AUCR_50_ (0.19)	38 37	6 6	31 31	4 5
Sundgren et al. [43]	13	Retro	46 (31-64)	54	WHO III: 9 WHO IV: 4	HGG with new enhan-cement after treatment	Histology (*N* = 5), imaging (*N* = 8)	1.5 T. T1 tra, sag 6/1.5 (470/min.); T1C tra, sag 6/1 · 5 (470/min.); T2 f. 6/1.5 mm (3000-5000/98); FLAIR tra 6/1.5 (10000/95/2200); DWI tra, cor, sag 6/0 (10000/min) b 0, 1000; DTI 4/0 (9300/min ms) b 0, 1000; MRS.	3-6 mo intervals; 28 mo after initial surgery	MRS (1.60-1.80)	7	0	6	0
Tie et al. [44]	19	Pros	51 (25-78)	63	WHO III: 12 WHO IV: 7	HGG with clinical or imaging suspicion of recurrence after treatment	Histology (*N* = 9), radioclinical (*N* = 10)	1.5 T. T1 tra -/- (-/-); T2 tra -/- (-/-); FLAIR tra -/- (-/-).	-	cMRI	11	1	3	6
Tsien et al. [45]	27	Pros	52 ± 3.1	-	WHO III: 4 WHO IV: 23	HGG after STR with min. 4 mL of residual tumour	Imaging (*N* = 27)	1.5-3 T. DSC - 4-6/0 (1500-2000/50-60).	Prior, 1 w after, 3 w after RT	rCBV	8	6	6	7
Yaman et al. [46]	17	Retro	45 (23-74)	65	WHO III: 2 WHO IV: 15	HGG with clinical or imaging suspicion of recurrence after treatment	Histology (*N* = 3), imaging (*N* = 14)	1.5 T. MRS multivoxel (-/35-135).	1 mo after CCRT + every 3 cycles of TMZ; 75% >6 mo post CCRT	MRS	13	0	4	0
Young et al. [47]	93	Retro	59 (9-84)	62	WHO IV: 93	GBM with new or enlarged enhan-cement after treatment	Histology (*N* = 28), imaging (*N* = 65)	1.5-3 T. T1C tra, cor, sag 5/0 (500/10); T2 tra 5/0 (4000-9000/100-125); FLAIR tra 5/0 (9000-10000/125-160/2200-2250).	4 weeks after end RT and with 1-2 mo intervals 1	cMRI	32	18	12	31
Zeng et al. [48]	26	Retro	40 ± 9.8 (23-65)	64	WHO III: 18 WHO IV: 6 WHO III/IV:4	HGG with new enhan-cement after treatment	Histology (*N* = 21), radioclinical (*N* = 5)	- T. T1 tra 6/- (-/-); T1C tra, cor, sag 6/- (-/-); T2 tra 6/- (-/-); FLAIR tra 6/- (-/-); MRS 3D 8 x 8 x 20-60 (1000/144).	6 w after RT end for MRI and 3-4 mo intervals	Cho/Cr (1.71) Cho/NAA (1.71)	16 15	0 0	9 9	1 2

The characteristics of the 35 included studies are shown. Abbreviations: ADC = apparent diffusion coefficient; cor = coronal; ASL = arterial spin labelling; AUC = area under the curve; BLS/PLD = baseline substraction/preload dosing; cat = category; CBV = cerebral blood volume; CCRT = concomitant chemoradiotherapy; cho = choline; cor = coronal; cMRI = conventional MRI; cre = creatine; d = days; DCE = dynamic contrast enhanced; DSC = dynamic susceptibility contrast; DWI = diffusion weighted imaging; DTI = diffusion tensor imaging; FA = Fractional anisotropy; FLAIR = fluid attenuation inversion recovery; FN = false negative; FP = false positive; fs = fat suppressed; GBM = glioblastoma multiforme; h = hours; HGG = high-grade glioma; K^trans^ = transfer constant between intra- and extracellular, extravascular space; NAA = N-acetyl-acetate; lac = lactate; mAUCR = mean area under the curve ratio; max = maximum; min = minimum; mm = millimetre; mo = months; MRS = magnetic resonance spectroscopy; ms = milliseconds; N = number; nCBV = normalised cerebral blood volume; PSR = percentage of signal intensity recovery; pros = prospective; PWI = perfusion weighted imaging; retro retrospective; rCBV = relative cerebral blood volume; ROC = Receiver operating characteristic; rPH = relative peak height; RT = radiotherapy; sag = sagittal; skew = skewness; STR = subtotal resection; SWI = susceptibility weighted imaging; T = Tesla; T1C = T1 post contrast; TE = echo time; TI = inversion time; TN = true negative; TP = true positive; TR = repetition time; tra = transversal; WHO = World Health Organisation; TMZ = temozolomide; w = weeks. * = in complete study cohort

Bivariate random effects models are used, because heterogeneity is to be expected in diagnostic test accuracy studies [49]. Pooled estimates of sensitivity, specificity, positive likelihood ratios, and negative likelihood ratios with 95%CI were calculated for each index test consisting of five or more studies, using the MIDAS module for meta-analysis of diagnostic test accuracy studies in STATA/SE 12.1 (College Station, TX, USA).

To provide insight in the potential clinical consequences, we established a hypothetical cohort of 100 HGG patients suggestive of progression for each MRI technique. We calculated 2x2 tables by using the mean tumour prevalence of the reference standard, pooled sensitivities and specificities of each MRI modality, and we present the number of misclassifications, false positives and false negatives. The hypothetical tumour prevalence was based on the mean tumour prevalence of the cohort studies included in this meta-analysis.

## Results

**Fig. 1:**
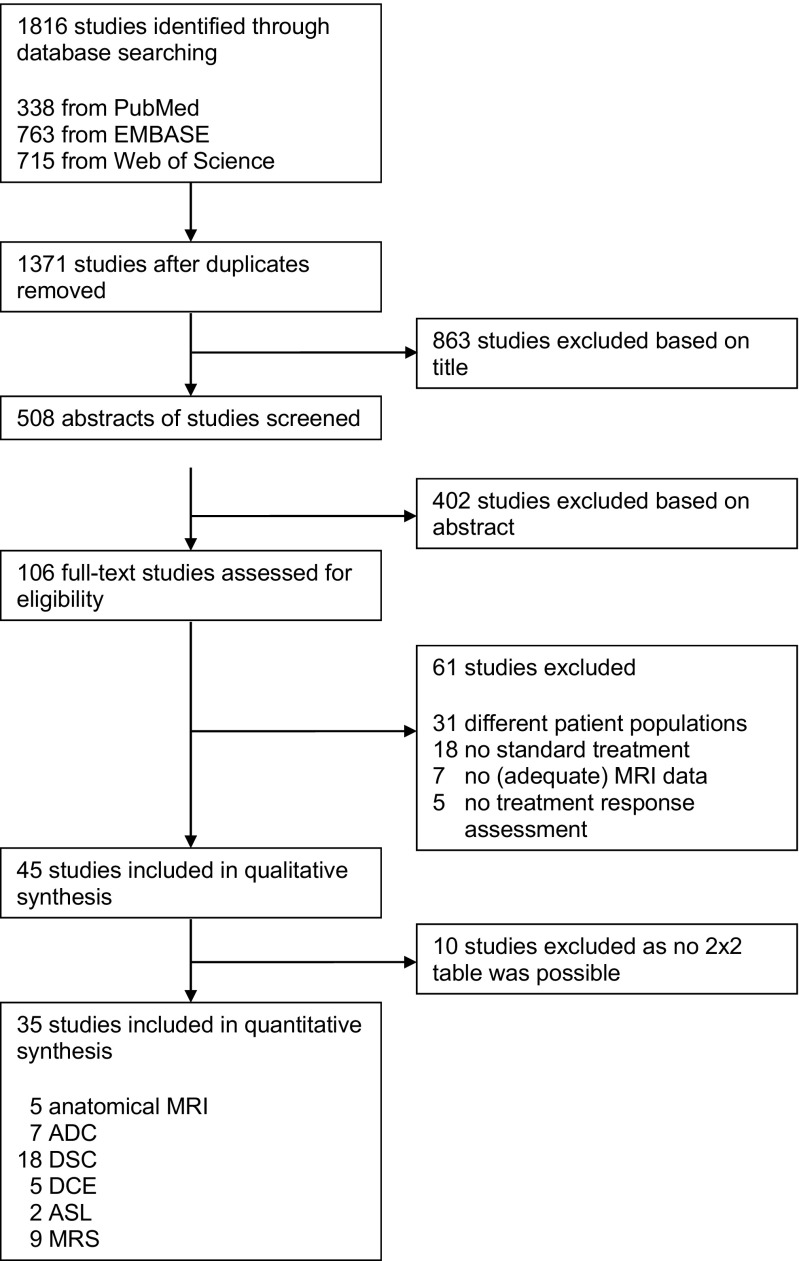
Flow chart of included studies. Flow chart of included studies. Abbreviations: ADC = apparent diffusion coefficient; ASL = arterial spin labelling; DCE = dynamic contrast enhanced; DSC = dynamic susceptibility contrast; MRI = magnetic resonance imaging; MRS = magnetic resonance spectroscopy

A total of 1371 unduplicated studies were identified through our electronic database search (Fig. 1). After selection based on title and abstract, the remaining studies underwent full-text eligibility assessment. Full text assessment resulted in the identification of 45 relevant studies [14–48, 50–59]. We requested data to generate 2x2 tables from ten studies, but none of the authors could provide the requested data, resulting in no unpublished data in this meta-analysis. Thus, final inclusion consisted of a total of 35 studies in this systematic review of which four (11%) were abstracts only [25, 26, 37, 38]. The study characteristics of the included and excluded studies are shown in Table 1 and Table 2, respectively.

**Table 2:** Characteristics of the excluded studies

Reference	*N*	Study type	Age (years) mean ± SD (range)	% male	Histology	Selection	Reference standard	Field strength; MRI techniques, orientation, slice thickness/ gap in mm (TR/TE/TI in ms); b values	Time point MRI	Diagnostic accuracy (cut-off)	TP	FP	TN	FN
Abel et al. [50] (abstract)	14	Retro	-	-	WHO IV: 14	GBM with new or enlarged enhan-cement fafter treatment	Imaging (*N* = 14)	- T. FLAIR	6-8 mo	Change in FLAIR volume				
Agerwal et al. [51]	46	Retro	57*	18*	WHO III: 6 WHO IV: 40	HGG with new or enlarged enhan-cement fafter treatment	Imaging (*N* = 46)	3 T. T1 tra 5/0 (3000/min); T2 tra 5/0 (3000/102); FLAIR tra 5/0 (10 · 000/120/2250); DTI 5/0 (8000-10000/84.3) b 0, 1000.	-	-				
Amin et al. [52]	19	Pros	55* (17-70)	54*	WHO III: 12 WHO IV: 7	HGG routine follow-up or with unclear cMRI or CT	Imaging (*N* = 19)	1.5 T. T1 tra, cor, sag 10-20/- (-/-); T1C tra, cor, sag10-20/- (-/-); T2 f. tra 10-20/- (-/-); FLAIR tra 10-20/- (-/-/-); MRS single voxel 4-8 cm^3^ (1500/30).	4-6 w after end of therapy	Cho/Cr Cho/NAA				
Fink et al. [53]	38	Retro	48 (28-70)	53	WHO III: 10 WHO IV: 12	HGG with suspected recurrence after treatment	Histology (*N* = 14), radioclinical (*N* = 26)	3 T. T1 tra 5/0 (400/10); T1C tra 5/0 (400/10); T2 tra 5/0 (3000/90); FLAIR tra (11.000/125/2800); DWI - 4/1 (5210/53), b 0, 1000; DSC tra 3/-(16/24); MRS multivoxel 10 x 10 x 12 (2000/144-288).	MRI after CCRT	CBV (2.08) ADC (1.28) Cho/Cr peak area (1.54) Cho/NAA peak height (1.05)				
Galldiks et al. [54]	25	Pros	54 (36-73)	60	WHO IV: 25	GBM patients undergoing surgery + CCRT	Imaging (*N* = 25)	1.5 T. T1 - 1/- (-/-); T2 - 1/- (-/-); FLAIR - 1/- (-/-).	11-20 d after surgery,7-10 d after end CCRT and 6-8 w after end CCRT	-				
Prat et al. [55]	20	Retro	-	58*	WHO III: 9 WHO IV: 11	HGG with new enhan-cement fafter treatment	Histology or multi-disciplinary consensus with imaging	- T. PWI - -/- (-/-); MRS - -/- (-/-).	After end CCRT	NAA/Cho (0.70)				
Shin et al. [56]	27	Retro	55* (27-72)	55*	WHO III: 7 WHO IV: 20	HGG with increased enhan-cement fafter treatment	Histology (*N* = 24), radioclinical (*N* = 7)	3 T. T1 - 5/- (250/3.5); T2 - 5/- (5500/93); FLAIR - 5/- (9000/95/ 2500); DCE - 4/- (4.3-5.1/1.5-1.8); DSC - 1.5/- (1880/30).	-	rCBV (2.33) rK^trans^ (2.1) AUC (2.29)				
Xu et al. [57]	31	Pros	45 (21-65)	54	WHO III: 14 WHO IV: 17	HGG with new enhan-cement fafter treatment	Histology (*N* = 23), imaging (*N* = 12)	3 T. T1 tra 5/1 (400/ 15);T2 tra 5/1 (3500/ 105); FLAIR tra 5/1 (10000/175/2200); DTI tra 5/1 (5000/97), b 0, 1000.	<72 h before re-resection or fbiopsy	ADC ratio (1.65) FA ratio (0.36)				
Xu et al. [58]	31	Pros	45 (21-65)	54	WHO III: 14 WHO IV: 17	HGG with new enhan-cement fafter treatment	Histology (*N* = 23), imaging (*N* = 12)	3 T. T1 tra 5/1 (400/ 15);T2 tra 5/1 (3500/ 105); FLAIR tra 5/1 (10000/175/2200); DSC tra 5/1 (1400/32).	-	rCBVmax (2.15)				
Zeng et al. [59]	55	Pros	44 (23-67)	55	WHO III: 36 WHO IV: 19	HGG with new enhan-cement fafter treatment	Histology (*N* = 39), imaging (*N* = 16)	3 T. T1 tra 6/- (500/8 ms); T1C tra, cor, sag 6/- (-/-); T2 tra 6/- (4500/102); FLAIR tra 6/- (9000/120/2250); DWI tra, cor, sag 6/- (5000/64 · 9), b 0, 1000; MRS multivoxel 10 x 10 x 10 (1500/144).	<6 w after end RT and with 2 mo intervals	Cho/Cr Cho/NAA ADC ratio				

The characteristics of the ten excluded studies are shown. For abbreviations see Table 1

The included studies consisted of 1174 patients with a mean age of 51.6 years of whom 61.3% were male (Table 3). The initial lesion was proven to be WHO type III in 11% (*N* = 124) and WHO type IV in 81% (*N* = 951). The remaining 8% (*N* = 99) was unspecified HGG. Mean tumour prevalence of the 34 cohort studies was 60% (range 31–85%). One case-control study was not taken into account for the calculation of the tumour prevalence [42]. Histological follow-up was used in 43% of patients (*N* = 502), imaging follow-up in 35% of patients (*N* = 406), clinical follow-up in <1% of patients (*N* = 3), and a combination of follow-up methods was used in 22% of patients (*N* = 263).

**Table 3:** General characteristics of included patients

Patients (*N*)	1174
Mean age (years)	51.6
% Male	61.3
Histology	
- WHO III	124
- WHO IV	951
- WHO III or IV (not specified)	99
Follow-up	
- Histology	502
- Imaging	406
- Clinical	3
- Combination	263

General characteristics are shown for the total of all included patients. See Table 1 for abbreviations.

Several of the included studies analysed multiple MRI modalities; therefore, a total of five anatomical MRI studies (*N* = 166) [23, 29, 39, 44, 47], seven apparent diffusion coefficient (ADC) studies (*N* = 204) [14, 15, 24, 25, 30, 33, 41], 18 dynamic susceptibility contrast (DSC) studies (*N* = 708) [15–17, 19, 20, 22–28, 30, 31, 37, 38, 40, 45], five studies on dynamic contrast enhanced (DCE) (*N* = 207) [18, 21, 32, 40, 42], two arterial spin labelling (ASL) studies (*N* = 102) [20, 40], and nine magnetic resonance spectroscopy (MRS) studies (*N* = 203) were included [22, 24, 34–36, 40, 43, 46, 48].

### Methodological quality of included studies

**Fig. 2:**
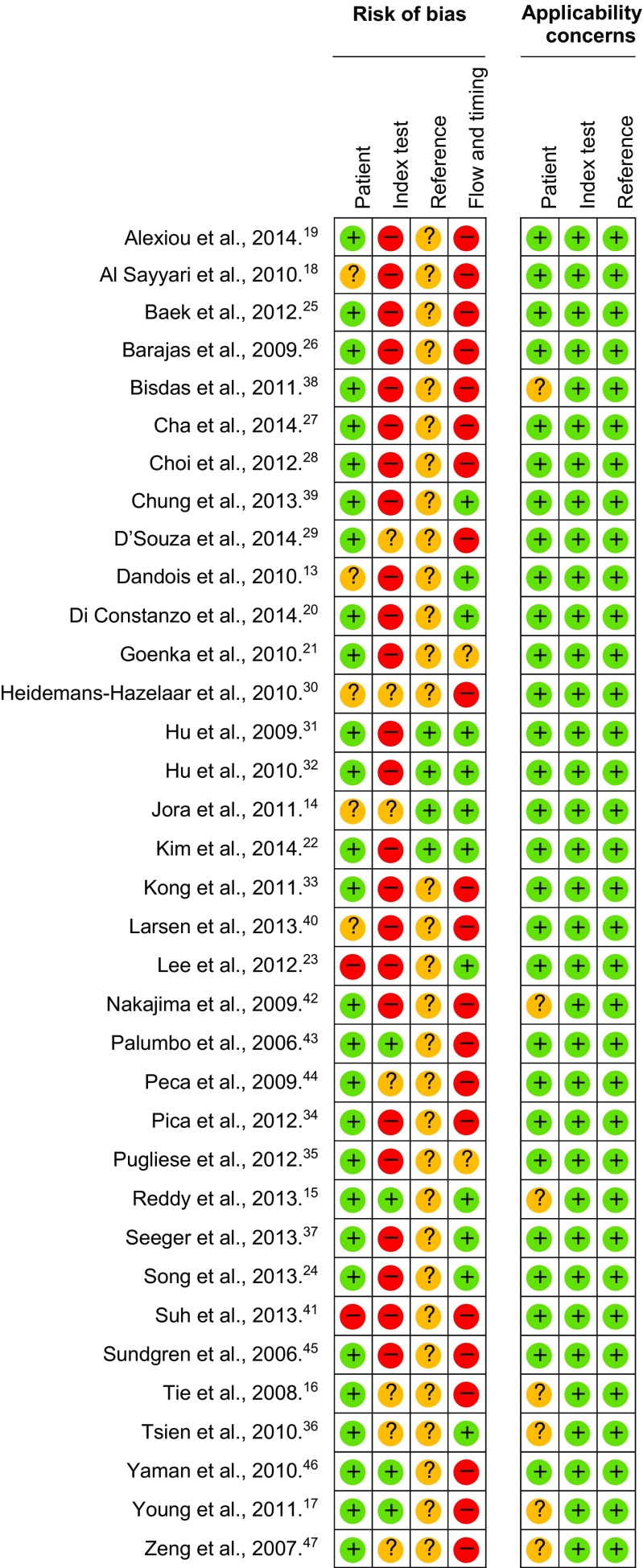
Quality assessment of included studies. The risk of bias in four different domains and concerns about applicability are shown for the included studies. High risk (), unclear risk () and low risk ()

See electronic supplementary material and Fig. 2.

### Main findings

**Fig. 3:**
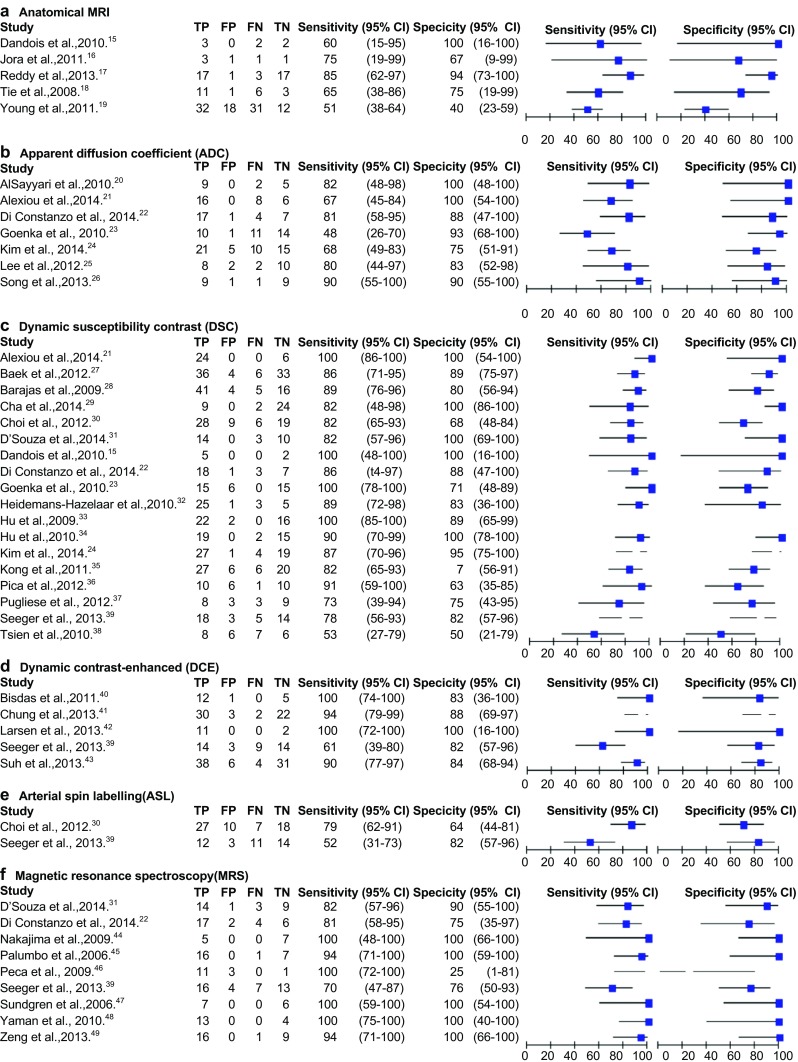
Forest plots with diagnostic accuracy of different MRI techniques. Diagnostic accuracy and the 2x2 table are displayed with true positives (TP), false positives (FP), false negatives (FN) and true negative (TN). Sensitivity and specificity with the 95% Confidence intervals (CI) are given

The forest plots and pooled results are demonstrated in Fig. 3 and Table 4, respectively. The anatomical MRI forest plot (five studies, 166 patients) shows a high variation in both sensitivity and specificity, with wide confidence intervals for three studies [23, 29, 44]. The wide confidence intervals of two references could be explained by the small sample size [23, 29]. The moderate methodological quality might explain the wider confidence intervals in the other study [44]. Anatomical MRI showed a pooled sensitivity and specificity of 68% (95%CI 51–81) and 77% (95%CI 45–93), respectively.

**Table 4:** Pooled accuracy of MRI techniques

	Studies	*N*	Sensitivity(95% CI)	Specificity(95% CI)	Positive LR (95% CI)	Negative LR (95% CI)
Anatomical MRI	5	166	68 (51-81)	77 (45-93)	2.9 (0.86-9.82)	0.42 (0.21-0.85)
ADC	7	204	71 (60-80)	87 (77-93)	5.4 (3.0-9.7)	0.33 (0.23-0.47)
DSC	18	708	87 (82-91)	86 (77-91)	6.1 (3.6-10.1)	0.15 (0.10-0.22)
DCE	5	207	92 (73-98)	85 (76-92)	6.4 (3.6-11.3)	0.09 (0.02-0.36)
MRS	9	203	91 (79-97)	95 (65-99)	17.2 (2.0-151.7)	0.09 (0.03-0.24)

Pooled diagnostic accuracy results are shown for all MRI sequences. Abbreviations: CI = confidence interval; LR = likelihood ratio; *N* = number. For other abbreviations see Fig. 1.

Sensitivity and specificity were both homogeneous in the forest plot of the ADC (seven studies, 204 patients); however, the confidence intervals are rather wide for the specificity. For ADC pooled sensitivity and specificity were 71% (95%CI 60–80), and 87% (95%CI 77–93), respectively. One abstract was included in this group [25], but sensitivity analysis excluding this study showed comparable sensitivity (75%, 95%CI 65–83) and specificity (85%, 95%CI 72–93) [15].

The sensitivity of the DSC (18 studies, 708 patients) is homogeneous with small confidence intervals. The specificity showed slightly more variability with wider confidence intervals. DSC showed a sensitivity of 87% (95%CI 82–91) and specificity of 86% (95%CI 77–91). This group included four abstracts [25, 26, 37, 38]. Sensitivity analysis excluding these studies showed minor increase in of the sensitivity with 87% (95%CI 81–92) and specificity of 89% (95%CI 80–95).

The confidence interval of the specificity of one study for the DCE (five studies, 207 patients) was also wide without clear reason [32], but the other studies showed small confidence intervals in both the sensitivity and specificity. For DCE the pooled sensitivity was slightly higher compared to the DSC with a sensitivity and specificity of 92% (95%CI 73–98) and 85% (95%CI 76–92), respectively.

For ASL, too few studies (two studies, 102 patients) were included in the meta-analysis for pooled accuracy estimate calculation. ASL showed a sensitivity range of 52–79% and a specificity range of 64–82%.

The forest plot of the MRS (nine studies, 203 patients) was overall homogeneous and showed small confidence intervals, with one exception in the specificity, possibly due to a moderate methodological quality as blinding was not assured both for the interpretation of the MRI as well as the reference standard [36]. MRS showed the highest pooled sensitivity and specificity with 91% (95%CI 79–97) and 95% (95%CI 65–99), respectively. Sensitivity analysis with the exclusion of one study [36] showed that it has only minor influences on the results altering the group sensitivity and specificity to 92% (95%CI 78–97) and 96 (95%CI 74–100).

Study design, mean age of patients, WHO type, cut-off value of the index test, type of follow-up, and time point of progression on MRI (see also Table 1) were evaluated as covariates and showed to be unable to explain differences in sensitivity and specificity of the studies.

To provide insight in the clinical implication of the investigated MRI techniques we also calculated the missed number of patients with true progression and total number of misclassifications in a hypothetical cohort of 100 HGG patients. We used the found tumour prevalence (60%) in this current analysis and the pooled sensitivity and specificity of each MRI technique. With anatomical MRI 19 recurrent tumours would be missed. For ADC and DSC this would be 17 and eight missed tumours, respectively. Both DCE and MRS would result in the least missed cases of progression (*N* = 5). Anatomical MRI would show a total of 28 misclassified patients. This would be 22, 14, and 11 for ADC, DSC, and DCE, respectively. MRS would induce the lowest number of misclassifications, with a total of seven out of the 100 patients being misclassified.

## Discussion

This meta-analysis including 35 studies, is the first pooling the results of all diagnostic MRI techniques in HGG patients following treatment. We demonstrated that all advanced MRI techniques showed a higher diagnostic accuracy than anatomical MRI in the differentiation between treatment induced changes and true progression. Among the advanced MRI techniques, MRS showed the highest diagnostic accuracy followed by perfusion MRI.

Diffusion derived ADC values showed the lowest accuracy of all advanced MRI techniques; however, it is currently most commonly available. We showed that the employment of novel advanced MRI techniques had higher diagnostic accuracy in the differentiation between true progression and treatment induced changes. Therefore, we suggest the incorporation of other advanced MRI in treatment assessment in HGG on top of DWI. This is supported by several studies that showed that diagnostic accuracy could significantly be enhanced by a combination of two or more advanced MRI techniques [60, 61]_._ Most important, adding MRS to perfusion weighted techniques could increase the diagnostic accuracy up to 90% in one study [40].

With a pooled sensitivity and specificity of 91% and 95%, respectively, we found MRS to be the most promising advanced MRI technique for the treatment response assessment in HGG. MRS, however, has several limitations. First, the voxel sizes are relatively large possibly leading to partial volume effects between recurrent tumour and treatment induced changes [4]. Detection of smaller lesions on MRS is, therefore, challenging. Secondly, due to low metabolite concentrations, a considerable number of acquisitions are required, resulting in long scan times [7]. Finally, MRS is technically challenging because of the need to exclude signal contamination from tissues adjacent to the tumour, such as lipids (from the scalp) and water (from the ventricles). Surgical clips also disrupt the local field homogeneity and may affect the quality of the data. These limitations challenge the incorporation of MRS in daily practice; however, a multivoxel technique should be feasible to perform in most clinics.

Various metabolic ratios were used in the MRS studies. In this meta-analysis we were unable to differentiate between the various metabolite ratios in MRS, because of the variability of the included ratios. Moreover, three of the included studies did not specify the investigated metabolite ratio [35, 43, 46]. However, five out of the nine included studies identified choline/creatine ratio as the best predictor in the differentiation between true progression and treatment induced changes [22, 24, 40, 43, 48]. One study reported similar results for choline/creatine and lactate/choline ratios, with the latter showing a slightly higher accuracy [34]. Furthermore, the included studies used various thresholds, or did not specify the used thresholds. Only one study used a considerably low cut-off value of 1.07, possibly explaining the low specificity of this study [40].

Among the perfusion techniques, DSC is the most widely used method. However, DSC is a dynamic parameter and values can vary over time. Yet, there is no consensus about the optimum time point. Furthermore, steroids are known to influence DSC measures, which are regularly prescribed if clinical deterioration due to true progression or treatment effects is present. Finally, there is no automatic post-processing method for identifying regions of interest, and is thus highly operator dependant [4]. This operator-dependant variability is also displayed in our meta-analysis by the different rCBV thresholds among studies (range 0.71–3.7).

DCE showed highest diagnostic accuracy among the perfusion techniques in the differentiation between treatment induced changes and true progression in this meta-analysis. At present, DCE is not widely used in a clinical setting primarily due to complicated quantification of the DCE parameters. Although DCE MRI has limited temporal resolution, the spatial resolution is higher than DSC MRI. This makes DCE more accurate in mixed lesions showing both true progression and treatment induced changes [7].

Although ASL is a complete non-invasive and quantitative method, the universal availability remains its largest limitation [8]. We could only identify two ASL studies and, therefore, it is not possible to make judgments reliably on the diagnostic accuracy of ASL in differentiating between true progression and treatment induced changes.

In our hypothetical cohort of 100 patients, ADC showed fewer misclassifications than anatomical MRI and could thus provide guidance to the definite diagnosis. ADC is a quantifiable measurement and can be achieved fast and easily [4]. However, the reliability of ADC can be affected by oedema and the formation of fibrosis in treatment induced changes [6].

A limitation that also should be noted is the inclusion of four abstracts. Inclusion of abstracts prevent a publication bias. However, quality and extend of information provided in abstracts is limited and they have not undergone the full peer review process as full articles.

Another possible limitation is that not all studies applied the same reference test. However, either histology or imaging follow-up was performed in all except three patients to provide definite diagnosis. Although we considered both histological follow-up and imaging follow-up to be reliable diagnostic methods, the reliability may not be equivalent. According to the Response Assessment in Neuro-Oncology (RANO) criteria, the development of pseudo-progression is limited to the first 3 months after CCRT [3]. However, it is suggested that 30% of pseudo-progression cases occur after more than three months post-CCRT [62]. Therefore, the accuracy of the reference test could differ between the included studies depending on the follow-up duration. However, no difference could be seen between early follow-up studies and studies that were conducted more than three months after CCRT.

Large multicentre longitudinal prospective trials are needed to define the optimum time for assessment of metabolic and physiological MRI parameters using advanced techniques. These should be in relation to histopathological changes in HGG, treatment effects, and patient outcomes. This would allow for testing all techniques in the same population, which would overcome one major limitation of the current meta-analysis with indirect comparisons only as a direct comparison between tests in a meta-analysis can only be performed if both contain >10 studies. These new prospective trials should use standardised cut-off values also, although they might remain arbitrary because of the heterogeneity in the biological activity of HGG and the use of different MRI systems. An advice with the best cut-off values and ratios for the anatomical and advance MRI sequences most precisely defining post therapy changes from tumour progression is currently hindered by the high variability of the used cut-offs and variables. However, it would be a valuable guideline for the clinician in daily practise. The latter could be addressed using normalised cut-off values. Despite these possible limitations, implication into clinical practice would be an important step in making an accurate treatment decisions for HGG patients.

## Conclusion

Our meta-analysis demonstrated a clear advantage of advanced MRI techniques for differentiation between true progression and treatment-induced changes in patients with HGG. All advanced MRI techniques showed a higher diagnostic accuracy than anatomical MRI. MRS showed the highest diagnostic accuracy followed by perfusion. Although a diffusion technique with ADC values is currently the most common used advanced technique, it showed the lowest diagnostic accuracy of all advanced MRI techniques. This study supports the extension of other advanced MRI techniques for assessment of treatment response in patients with HGG.

## Electronic supplementary material

Below is the link to the electronic supplementary material.

ESM 1 (DOCX 20 kb)

ESM 2 (DOCX 16 kb)

## Abbreviations

ADC: Apparent diffusion coefficient
ASL: Arterial spin labelling
CCRT: Concomitant chemoradiotherapy
CI: Confidence interval
DCE: Dynamic contrast enhanced
DSC: Dynamic susceptibility contrast
HGG: High-grade glioma
MRS: Magnetic resonance spectroscopy
PRISMA: Preferred reporting items for systematic reviews and meta-analysis
QUADAS: Quality assessment of diagnostic accuracy studies
RANO: Response assessment in neuro-oncology
rCBV: Relative cerebral blood volume
TMZ: Temozolomide
WHO: World Health Organisation
