# Supplementary material for: Diagnostic accuracy of magnetic resonance imaging techniques for treatment response evaluation in patients with high-grade glioma, a systematic review and meta-analysis
Source: Eur Radiol. 2017 Mar 22;27(10):4129–44. doi: 10.1007/s00330-017-4789-9 (PMC5579204; doi:10.1007/s00330-017-4789-9)
Supplement: Supplementary file 1 — (DOCX 20 kb) [file 330_2017_4789_MOESM1_ESM.docx]

**SUPPLEMENTARY MATERIAL**

**Search strategy**

We searched PubMed, EMBASE, and Web of Science using a search strategy consisting of database keywords and text words, with the latest search on February 5th, 2015. Our search term was composed to describe glioma, MR techniques, and treatment evaluation and variations of these words (see web extra material). No filters were used although studies written in other languages than English were excluded manually. Grey literature was also included in the search as EMBASE contains conference proceedings.

**Search strategy PubMed:**

("Glioma"[Mesh] OR glioma[tw] OR glioblastom*[tw] OR astrocytom*[tw] OR oligodendrogliom*[tw] OR oligoastrocytom*[tw])

**AND** (“magnetic resonance imaging”[MesH] OR MRI [tw] OR magnetic resonance[tw] OR MR imaging[tw] OR diffusion[tw] OR DWI[tw] OR apparent diffusion coefficient[tw] OR ADC[tw] OR perfusion[tw] OR dynamic contrast[tw] OR contrast enhanced[tw] OR "dynamic susceptibility contrast"[tw] OR DSC[tw] OR “spectroscopy”[tw] OR MRS[tw] OR nuclear magnetic resonance[tw] OR NMR[tw] OR chemical shift imaging[tw] OR blood-oxygen-level dependent[tw] OR BOLD[tw])

**AND** ((“disease progression”[MeSH] OR differentiat*[tw] OR differential[tw] OR discriminat*[tw] OR disting*[tw] OR distinc*[tw] OR response[tw] )

**AND** (treatment-induced[tw] OR radiation induced[tw] OR radiation associat*[tw] OR radiation chang*[tw] OR radiation effect*[tw] OR treatment effect*[tw] OR posttreatment[tw] OR post treatment[tw] OR posttherapeutic[tw] OR post therapeutic[tw] OR post irradiation[tw] OR postirradiation[tw] OR post radiation[tw] OR postradiation[tw] OR treatment outcome[tw] OR residual tumour[tw] OR residual tumor[tw] OR radiation injur*[tw] OR pseudo progression[tw] OR pseudoprogression[tw] OR radiation necrosis[tw] OR radio necrosis[tw] OR tumour progression[tw] OR tumor progression[tw] OR disease progression[tw] OR recurrent tumour[tw] OR recurrent tumor[tw] OR tumour recurrence [tw] OR tumor recurrence [tw] OR true tumor[tw] OR true tumour[tw] OR treatment-related[tw]))

**Search strategy EMBASE:**

(“glioma”/exp OR “astrocytoma”/exp OR “glioma”:ab,ti OR glioblastom*:ab,ti OR astrocytom*:ab,ti OR oligodendrogliom*:ab,ti OR oligoastrocytom*:ab,ti)

**AND** (“nuclear magnetic resonance imaging”/exp OR “nuclear magnetic resonance”/exp OR “spectroscopy”/exp OR “BOLD signal”/exp OR MRI:ab,ti OR “magnetic resonance”:ab,ti OR “MR imaging”:ab,ti OR “diffusion”:ab,ti OR DWI:ab,ti OR “apparent diffusion coefficient”:ab,ti OR ADC:ab,ti OR “perfusion”:ab,ti OR “dynamic contrast”:ab,ti OR “contrast enhanced”:ab,ti OR “dynamic susceptibility contrast”:ab,ti OR DSC:ab,ti OR “spectroscopy”:ab,ti OR MRS:ab,ti OR “nuclear magnetic resonance”:ab,ti OR NMR:ab,ti OR “chemical shift imaging”:ab,ti OR “blood-oxygen-level dependent”:ab,ti OR BOLD:ab,ti)

**AND** (“differentiation”/exp OR differentiat*:ab,ti OR “differential”:ab,ti OR discriminat*:ab,ti OR disting*:ab,ti OR distinc*:ab,ti OR “response”:ab,ti)

**AND** (“radiation response”/exp OR “treatment outcome”/exp OR “minimal residual disease”/exp OR “brain necrosis”/exp OR “radiation injury”/exp OR “disease course”/exp OR “disease progression”:ab,ti OR “treatment-induced”:ab,ti OR “radiation induced”:ab,ti OR “radiation associated”:ab,ti OR “radiation association”:ab,ti OR “radiation change”:ab,ti OR “radiation changes”:ab,ti OR “radiation effect”:ab,ti OR “radiation effects”:ab,ti OR “treatment effect”:ab,ti OR “treatment effects”:ab,ti OR “posttreatment”:ab,ti OR “post treatment”:ab,ti OR “posttherapeutic”:ab,ti OR “post therapeutic”:ab,ti OR “post irradiation”:ab,ti OR “postirradiation”:ab,ti OR “post radiation”:ab,ti OR “postradiation”:ab,ti OR “treatment outcome”:ab,ti OR “residual tumour”:ab,ti OR “residual tumor”:ab,ti OR “radiation injury”:ab,ti OR “radiation injuries”:ab,ti OR “pseudo progression”:ab,ti OR “pseudoprogression”:ab,ti OR “radiation necrosis”:ab,ti OR “radio necrosis”:ab,ti OR “tumour progression”:ab,ti OR “tumor progression”:ab,ti OR “disease progression”:ab,ti OR recurrent tumour:ab,ti OR recurrent tumor:ab,ti OR tumour recurrence:ab,ti OR tumor recurrence:ab,ti OR “true tumour”:ab,ti OR “true tumor”:ab,ti OR “treatment-related”:ab,ti)

**Search strategy web of science:**

(TS=(“glioma” OR “gliomas” OR “glioblastoma” OR “glioblastomas” OR “astrocytoma” OR “astrocytomas” OR “oligodendroglioma” OR “oligodendrogliomas” OR “oligoastrocytoma” OR “oligoastrocytomas”))

**AND** (TS=(“magnetic resonance imaging” OR “MRI” OR “magnetic resonance” OR “MR imaging” OR “diffusion” OR “DWI” OR “apparent diffusion coefficient” OR “ADC” OR “perfusion” OR “dynamic contrast” OR “contrast enhanced” OR "dynamic susceptibility contrast" OR “DSC” OR “spectroscopy” OR “MRS” OR “nuclear magnetic resonance” OR “NMR” OR “chemical shift imaging” OR “blood-oxygen-level dependent” OR “BOLD”))

**AND** (TS=(“differentiating” OR “differentiated” OR “differential” OR “discriminating” OR “discrimination” OR “discriminated” OR “distinguishing” OR “distinguished” OR “distinguish” OR “distinction” OR “response” OR “treatment-induced” OR “radiation induced” OR “radiation associated” OR “radiation association” OR “radiation change” OR “radiation changes” OR “radiation effect” OR “radiation effects” OR “treatment effect” OR “treatment effects” OR “posttreatment” OR “post treatment” OR “posttherapeutic” OR “post therapeutic” OR “post irradiation” OR “postirradiation” OR “post radiation” OR “postradiation” OR “treatment outcome” OR “residual” OR “necrosis” OR “necrotic” OR “radiation injury” OR “radiation injuries” OR “pseudo progression” OR “pseudoprogression” OR “radiation necrosis” OR “radio necrosis” OR “tumour progression” OR “tumor progression” OR “disease progression” OR “recurrent” OR “recurrence” OR “true” OR “stable” OR “treatment-related”))
