# Supplementary material for: Diagnostic accuracy of magnetic resonance imaging techniques for treatment response evaluation in patients with high-grade glioma, a systematic review and meta-analysis
Source: Eur Radiol. 2017 Mar 22;27(10):4129–44. doi: 10.1007/s00330-017-4789-9 (PMC5579204; doi:10.1007/s00330-017-4789-9)
Supplement: Supplementary file 2 — (DOCX 16 kb) [file 330_2017_4789_MOESM2_ESM.docx]

**SUPPLEMENTARY MATERIAL**

**Methodological quality of included studies**

In the first domain regarding patient selection, three out of 35 studies (9%) were considered to be of high risk of bias [25,43,38]. Two of these studies showed inappropriate exclusion criteria as patients with definite disease progression according to RANO or histological confirmation were not taken into account [25], or patients who underwent gross-total resection were excluded [38]. Both might induce a selection bias. One study did not avoid a case-control design and was, therefore, also considered to be of high risk of bias [43]. Another five studies (14%) were considered to be of unclear risk of bias due to concerns about whether the patient enrolment was random or consecutive, because in these studies patients with residual tumour or patients with clinical deterioration were included [15,16,20,32,42]. The remaining (77%) were considered to be of low risk of bias [17–19,21–24,26–31,33–37,39–41,44–49].

In the index test domain 24 studies (69%) did not specify the used MRI threshold or cut-off value and were, therefore, considered to be of high risk of bias [15,20–30,33–37,39–44,47]. In an additional seven studies (20%) it was not assured that the results of the reviewed MRI technique were interpreted without knowledge of the results of the reference standard [16,18,31,32,38,46,49]. Hence, we considered them to be of unclear risk of bias. We considered the four remaining studies (11%) to be of low risk [17,19,45,48].

In the domain of the reference standard, no studies were considered to be of high risk. In 31 studies (89%), it was unclear if the results of the reference test were interpreted without knowledge of the index test [15,17–23,25–32,35–49]. All four remaining studies (11%) were considered to be of low risk of bias [16,24,33,34].

Finally, in the flow and timing domain, 21 studies (60%) were considered to be of high risk of bias, because not all patients received the same reference standard [18–21,27–32,35,36,40,42–49]. Two studies (6%) did not specify the interval between the index test and reference standard and were, therefore, regarded to have an unclear risk of bias [23,37]. The other 12 studies (34%) showed a low risk of bias [15–17,22,24–26,33,34,38,39,41].

All studies showed high risk of bias in at least one of the four domains with the exception of two studies. One study was considered to be of low risk of bias in all domains except the reference standard domain where it showed an unclear risk [15]. Another study was considered to be of low risk of bias in two out of four domains, but showed an unclear risk in the patient selection domain and the index text domain [14]. Overall, study quality can therefore be regarded as moderate.

Regarding the applicability assessment, we had limited concerns that the included patients and setting matched our review question in six studies [17–19,38,40,49]. In one study, patients <18 years of age (range 9–84) were included in the cohort of patients [19]. For an additional five studies we had concerns regarding the received therapy. In one case the authors did not mention the administration of chemotherapy [49]. In four studies, other chemotherapy agents besides TMZ, such as bevacizumab [40], carmustine [17,18], and gemticitabine [38], were also administered in a minority of patients (5–15%). We had no concerns that the conduct and interpretation of the index test, and the reference standard did not match our review question in any of the studies. In conclusion, we had no applicability concern for 29 out of the 35 included studies [15,16,20–37,39,41,42,43–48].
